# Factors associated with increased risk of lurasidone-induced somnolence: Two case-control studies based on one bioequivalence trial in healthy volunteers

**DOI:** 10.1016/j.heliyon.2023.e17905

**Published:** 2023-07-01

**Authors:** Hengyi Yu, Xingxing Qi, Yinian Fang, Kaifu Wang, Donglin Zhang, Qian Chen, Dong Liu, Xiuhua Ren

**Affiliations:** Department of Pharmacy, Tongji Hospital, Tongji Medical College, Huazhong University of Science and Technology, Hubei Wuhan, 430030, China

**Keywords:** Lurasidone, Somnolence, Plasma concentration, Healthy volunteer, Case-control study

## Abstract

Somnolence is a common adverse effect of antipsychotic drugs used to treat psychotic disorders. It causes problems in many areas of life, such as gainful employment, driving, childcare, and social interactions. Somnolence is a major problem for a relatively new antipsychotic drug, lurasidone, whose dose-effect relationship remains unclear. Based on data from a bioequivalence study of two 40 mg lurasidone hydrochloride tablets, we designed two case-control studies to explore the correlation between somnolence and exposure to lurasidone and determine the factors associated with lurasidone-induced somnolence. In the first case-control study, lurasidone was administered to healthy volunteers; 30 experienced somnolence (as pre-defined) but 29 did not. Moreover, plasma concentration at 1 h was significantly associated with somnolence (OR = 1.124; p = 0.001). In the second case-control study, 48 volunteers administered lurasidone were classified into somnolence and no-somnolence groups based on different time-related criteria. We observed a positive association between plasma concentration at 0.75 h and somnolence (OR = 1.024; p = 0.002). Receiver operating characteristic analysis revealed that a plasma lurasidone concentration >21.65 ng/mL 1 h after administration strongly predicted somnolence. Our findings in healthy volunteers need to be further validated in patients in clinical settings to determine the optimal dose and duration of lurasidone administration.

## 1 Introduction

Schizophrenia is a chronic and severe mental disorder that affects the thoughts, feelings, and behaviors of the patients. It affects approximately 20 million people worldwide, and patients with schizophrenia typically exhibit long-term psychosocial impairments that affect their education and employment opportunities [1]. Administration of antipsychotics is the standard treatment for schizophrenia.

Lurasidone is a second-generation antipsychotic that was approved by the US Food and Drug Administration in 2010 for the treatment of schizophrenia and bipolar depression in adults and adolescents [2] and by the European Medicines Agency in 2014 for the treatment of schizophrenia in adults [3]. Lurasidone was the first atypical antipsychotic approved for the treatment of schizophrenia in adolescents aged 13–17 years in Europe in 2020 [4]. In addition to its principal antagonistic activity against dopamine D2 and serotonin 5-HT2A receptors, lurasidone also exerts antagonistic activity against 5-HT7. It exhibits partial agonism for 5-HT1A receptors and modest antagonism for noradrenergic a2A and a2C receptors [5] but no affinity for histamine H1 or acetylcholine M1 receptors due to its unique chemical structure [6]. Based on its pharmacological profile, lurasidone is associated with fewer extra-pyramidal symptoms than conventional agents and effectively ameliorates various schizophrenic symptoms [5,7]. Lurasidone has a lower risk of weight gain or metabolic disturbances than other commonly used antipsychotics [8,9]. Based on the effect of concomitant food intake on the bioavailability of lurasidone [10], dosing instructions recommend the use of lurasidone with a meal of at least 350 kcal caloric value to optimize its relevant pharmacokinetic parameters [7].

Somnolence is one of the most commonly lurasidone-induced adverse reactions in patients, and its incidence varies depending on the individual, symptom severity, dosage, and other circumstantial factors. In some short-term, placebo-controlled schizophrenia studies, somnolence was reported in 17.0% of 1508 patients treated with lurasidone [2]. This seemingly harmless side effect may be beneficial to patients with acute psychosis, agitation, mania, or insomnia; however, it can be harmful and cause various serious consequences, such as decreased cognitive and motor performance and increased risk-taking behavior, in sensitive patients [11]. Severe somnolence, especially in outpatient settings, can cause accidents, difficulties in school, loss of employment, interpersonal problems, and neglect of oneself or others [11]. Somnolence is one of the most common side effects responsible for the premature discontinuation of acute treatment in patients with bipolar depression, major depressive disorder, and generalized anxiety disorder [12,13]. Mothers with schizophrenia risk losing custody of their infants if they are perceived as potentially neglectful because of excessive daytime sleepiness [14]. Although bedtime administration of lurasidone with food has been recommended [8,10,13], it may not be feasible for all patients, especially those with irregular bedtime and mealtime routines.

Incidence of somnolence has been positively correlated with the dose and duration of some antipsychotics, but not for others [11]. Although the dosage of lurasidone can affect the incidence of somnolence, their dose-effect relationship remains unclear. In some studies, the rate of somnolence increased as the drug dose increased [[15], [16], [17], [18], [19]], but this trend was not evident in other studies [20,21]. These inconsistent results motivated us to explore the relationship between somnolence and lurasidone exposure and determine the factors associated with lurasidone-induced somnolence in this study. We designed and conducted two independent case-control studies in this work based on the data of one bioequivalence study.

## 2 Materials and methods

### 2.1 Bioequivalence study

The bioequivalence study was a randomized, two-way crossover, single-dose, two-period study to evaluate the bioavailability of two lurasidone hydrochloride tablets (40 mg): a test preparation manufactured by a domestic company and a reference preparation (Latuda®) manufactured by Sumitomo Dainippon Pharma Co., Ltd. The bioequivalence study (chinadrugtrials.org identifier: CTR20191783) was approved by the Drug Clinical Trial Ethics Committee of Huazhong University of Science and Technology and conducted at the Tongji Hospital according to the Declaration of Helsinki and the International Council for Harmonisation of Technical Requirements for Pharmaceuticals for Human Use Guideline for Good Clinical Practice. Written informed consent was obtained from all volunteers.

**Fig. 1:**
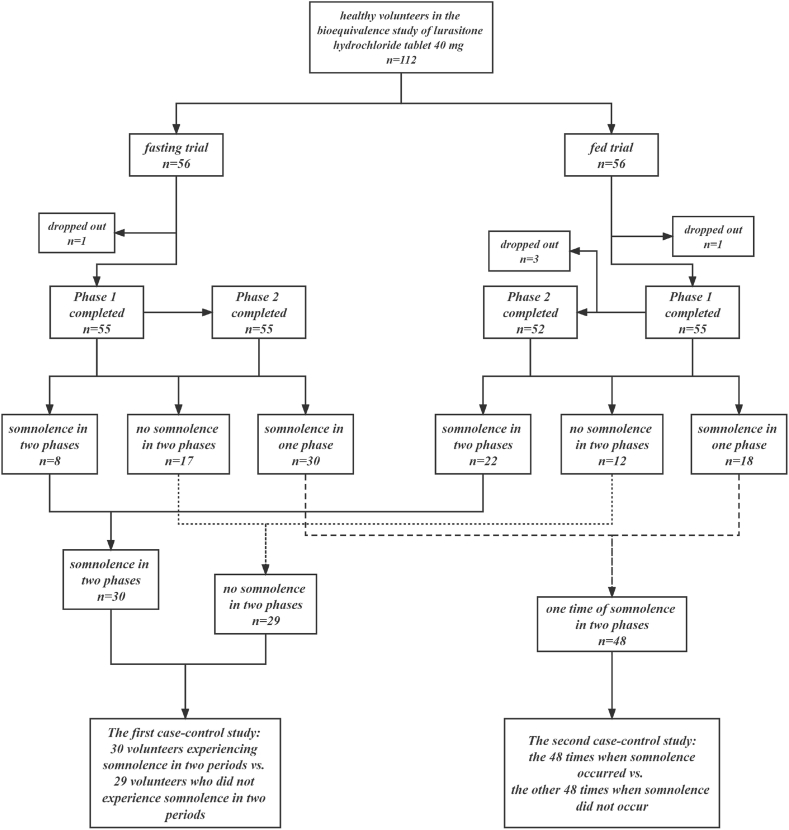
Flow chart of the two independent case-control studies designed based on one bioequivalence study.

As shown in Fig. 1, in the bioequivalence study, fasting and fed trials were conducted in two different groups of volunteers. Healthy volunteers were randomly assigned to two groups (1:1) and received a single dose of either the test or reference preparation of 40 mg lurasidone hydrochloride tablets and a single dose of the alternate preparation after a seven-day washout period. Blood samples (4 mL) were collected from the subjects at time 0 (pre-dosing) and other time points (0.25, 0.5, 0.75, 1, 1.5, 2, 2.5, 3, 3.5, 4, 5, 6, 8, 12, 24, 36, and 48 h) after drug administration and centrifuged (1500×*g*, 4 °C, 10 min) to obtain plasma, which was stored at −70 °C until analysis.

Plasma concentration of lurasidone was determined by a verified LC-MS/MS method following a reported method [22] with minor modification. Calibration curve ranged from 0.2 to 100 ng/mL. Main pharmacokinetic parameters of the test (T) and reference (R) preparations were calculated using the Phoenix WinNonlin 7.1 software. Under fasting conditions: for the two preparations, the maximum concentrations (C_max_) were 26.1 and 24.1 ng/mL, respectively, area under the concentration-time curve from time 0 to time t (AUC_0-t_) values were 109 and 100 h × ng/mL, respectively, and area under the curve from time 0 extrapolated to infinite time (AUC_0-∞_) values were 116 and 107 h × ng/mL, respectively. Moreover, the 90% confidence intervals (CIs) of C_max_, AUC_0-t_, AUC_0-∞_ between T and R were 96.61–121.13, 102.88–114.59, and 102.78–113.96%, respectively. Under fed conditions: C_max_ values were 70.0 and 74.6 ng/mL, respectively, AUC_0-t_ values were 252 and 259 h × ng/mL, respectively, and AUC_0-∞_ values were 270 and 278 h × ng/mL, respectively. Furthermore, the 90% CIs of C_max_, AUC_0-t_, and AUC_0-∞_ between T and R were 87.10–100.95, 93.66–101.17, and 93.23–100.99%, respectively. Consequently, the test preparation was bioequivalent to the reference preparation, which has been approved by the National Medical Products Administration of China [23].

During the bioequivalence study, any adverse events observed by the investigators or self-reported by the volunteers were carefully monitored, evaluated, treated if necessary, and recorded by a clinical research physician using the Common Terminology Criteria for Adverse Events (CTCAE) Version 5.0 [24]. For somnolence assessment, the Stanford Sleepiness Scale (SSS), which is a self-rating scale used to quantify the subjective changes in sleepiness, was used in the bioequivalence study. SSS is based on a single item rated on a Likert-type scale from 1 (feeling active and vital, alert, or wide awake) to 7 (almost in reverie, sleep onset soon, or lost the struggle to remain awake), with higher scores indicating more subjective sleepiness [25]. If a volunteer got a score of 7 after lurasidone administration during daytime, the clinical research physician determined that somnolence occurred.

### 2.2 Study design and setting

Based on the occurrence of somnolence in the bioequivalence study, two independent case-control studies were carefully designed to further evaluate the existing data. The first case-control study was conducted to compare the healthy volunteers experiencing somnolence during the two test periods (somnolence group) with those who did not experience somnolence during either test period (no-somnolence group). The second case-control study was conducted with the same volunteers; however, somnolence or no-somnolence was defined as testing positive or negative during one (rather than two) testing periods, respectively.

### 2.3 Data collection

Two researchers extracted the following data from the volunteer medical records for the bioequivalence study: demographic information (gender, age, height, and weight), study information (fasting/fed conditions and type of preparation in each period), and somnolence (occurring or not, starting time, ending time, severity, and causality). The plasma concentrations of lurasidone at each time point and the main calculated pharmacokinetic parameters related to exposure (C_max_, T_max_, and AUC_0-∞_) were extracted from the pharmacokinetic study report.

### 2.4 Statistical analysis

All analyses were performed using IBM SPSS Statistics (version 23.0; IBM, Armonk, NY). Distribution of continuous data was tested using the Kolmogorov-Smirnov test. Normally distributed variables were expressed as the mean ± standard deviation and compared using an independent-sample *t*-test. Non-normally distributed data were expressed as the median (interquartile range) and compared using the Mann-Whitney *U* test. Categorical variables were reported as numbers (percentages) and compared using the chi-square or Fisher's exact test. Type I error rate for significance tests between the somnolence and no-somnolence groups was set at α = 0.05 (two-tailed). Univariate and multivariate odds ratios (ORs) for the independent variables and somnolence were calculated using logistic regression models and presented as ORs with 95% CIs to reflect the magnitude of variance. Independent variables with p ≤ 0.1 for association with the response variable in the univariable analysis were tested in the multivariable model. For independent variables, statistical significance was declared when the CI did not include zero. Receiver operating characteristic curves were used to evaluate the predicted probabilities for the different models. For all tests, statistical significance was set at p < 0.05.

## 3 Results

### 3.1 Somnolence in the bioequivalence study

As shown in Figs. 1 and 112 volunteers participated in the bioequivalence study: 56 each in the fasting and fed trials. During the bioequivalence study, two volunteers dropped out in Period 1, and three in Period 2. Finally, 107 volunteers took lurasidone hydrochloride tablets and completed the study in two periods. Among the 107 volunteers, 30 experienced somnolence in both periods, 29 did not experience any somnolence in either period, and 48 experienced somnolence in only one of the two periods.

According to the CTCAE [24], the severity of somnolence occurred in the bioequivalence study was all classified as ‘grade 1’ by the clinical research physician, which was mild but more than usual drowsiness or sleepiness. Only clinical observations were conducted and symptoms of somnolence were relieved without intervention after a median lasting time of 1.05 (0.58, 1.68) hours. The causality category of somnolence was classified as ‘certain’ in 30 case-times, and as ‘probable/likely’ in 78 case-times [26].

### 3.2 Differences in variables between the somnolence and no-somnolence groups

**Fig. 2:**
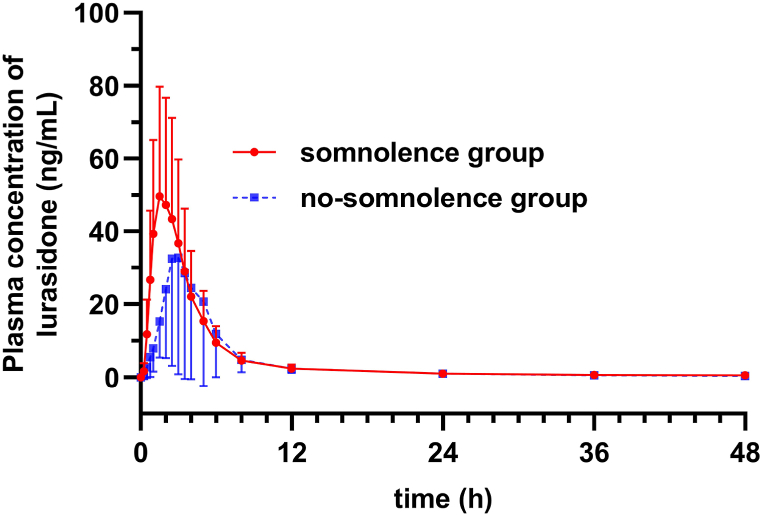
Mean plasma concentration-time curves of lurasidone in the somnolence (n = 30 volunteers) and no-somnolence (n = 29 volunteers) groups of the first case-control study.

In the first case-control study, 30 volunteers who experienced somnolence in the two periods were classified into the somnolence group, and 29 volunteers who did not experience somnolence in the two periods were classified into the no-somnolence group. As shown in Table 1, no significant differences were found in gender, age, height or weight between the two groups. However, there was a significantly higher proportion of volunteers under fed condition in the somnolence group than in the no-somnolence group (73.3 vs. 41.4%; p = 0.013). Moreover, the median C_max_ (64.90 vs. 26.75 ng/mL; p = 0.004) and AUC_0-∞_ (236.25 vs. 127.00 ng × h/mL; p = 0.038) values in the somnolence group were significantly higher, whereas the median T_max_ (1.75 vs. 2.50 h; p = 0.001) values were significantly lower in the somnolence group than in the no-somnolence group. Pharmacokinetic characteristics of the two groups are shown in Fig. 2.

**Table 1:** Variables in the somnolence (n = 30 volunteers) and no-somnolence (n = 29 volunteers) groups of the first case-control study.

Variables	Total volunteers (*n* = 59)	Somnolence group (*n* = 30)	No-somnolence group (*n* = 29)	statistics	p-value
Gender				χ^2^ = 0.060	0.807
Female	13 (22.0%)	7 (23.3%)	6 (20.7%)		
Male	46 (78.0%)	23 (76.7%)	23 (79.3%)		
Age (y)	28.0 (22.0, 33.0)	25.0 (21.0, 31.2)	29.0 (23.0, 36.0)	*Z* = −1.732	0.083
Height (m)	1.70 (1.60, 1.75)	1.70 (1.58, 1.76)	1.70 (1.62, 1.75)	*Z* = −0.144	0.885
Weight (kg)	63.79 ± 8.36	63.51 ± 9.05	64.08 ± 7.72	*t* = 0.260	0.796
Fed condition				χ^2^ = 6.166	0.013*
Fasting	25 (42.4%)	8 (26.7%)	17 (58.6%)		
Fed	34 (57.6%)	22 (73.3%)	12 (41.4%)		
C_max_ (ng/mL)	55.05 (22.85, 92.80)	64.90 (45.90, 96.79)	26.75 (15.65, 70.50)	*Z* = −2.881	0.004*
T_max_ (h)	2.00 (1.50, 3.00)	1.75 (1.25, 2.31)	2.50 (2.00, 3.25)	*Z* = −3.355	0.001*
AUC_0-∞_ (ng × h/mL)	201.50 (124.50, 305.50)	236.25 (164.13, 298.25)	127.00 (82.70, 312.25)	*Z* = −2.077	0.038*

*p < 0.05.

**Fig. 3:**
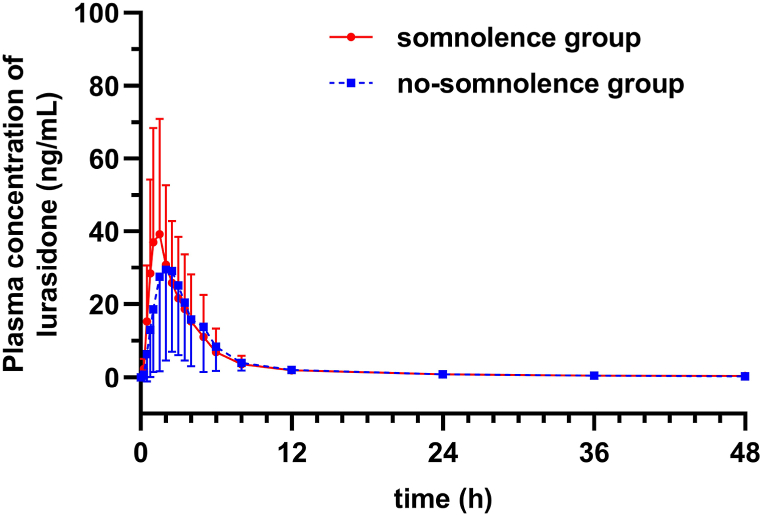
Mean plasma concentration-time curves of lurasidone in the somnolence (n = 48 times) and no-somnolence (n = 48 times) groups of the second case-control study.

In the second case-control study, 48 volunteers experienced only one time of somnolence in two periods, yielding 96 sets of the main calculated pharmacokinetic parameters. Therefore, the 48 times when somnolence occurred were taken into somnolence group, and the remaining 48 times when somnolence did not occur were taken into the no-somnolence group. As volunteers in the somnolence and no-somnolence groups were the same individuals, the demographic information (gender, age, height, and weight) and food intake conditions were identical for both groups. Among the main calculated pharmacokinetic parameters, only the median T_max_ (1.5 vs. 2.0 h; p = 0.028) value was significantly lower in the somnolence group than in the no-somnolence group. No significant differences in preparation, C_max_ and AUC_0-∞_ values were observed between the two groups (p > 0.05) (Table 2). Pharmacokinetic characteristics of the two groups are shown in Fig. 3.

**Table 2:** Variables in the somnolence (n = 48 times) and no-somnolence (n = 48 times) groups of the second case-control study.

Variables	Total times (*n* = 96)	Somnolence group (*n* = 48)	No-somnolence group (*n* = 48)	statistics	p-value
Preparations				χ^2^ = 0.000	1.0
Test	48 (50%)	24 (50%)	24 (50%)		
Reference	48 (50%)	24 (50%)	24 (50%)		
C_max_ (ng/mL)	39.6 (27.00, 66.08)	46.80 (27.90, 71.77)	38.25 (23.22, 63.38)	*Z* = −1.704	0.088
T_max_ (h)	1.5 (1.0, 2.5)	1.5 (1.0, 2.00)	2.0 (1.5, 2.5)	*Z* = −2.199	0.028*
AUC_0-∞_ (ng × h/mL)	161.5 (108.5, 248.75)	162.50 (113.75, 247.50)	141.50 (92.75, 251.00)	*Z* = −0.458	0.647

*p < 0.05.

Although the difference in variables between the somnolence and no-somnolence groups in the two case-control studies were not exactly the same, the median T_max_ values of both somnolence groups in the two studies were very close (1.75 and 1.5 h) and lower than those of the no-somnolence groups.

### 3.3 Associations between independent variables and somnolence

As C_max_, AUC_0-∞_, and T_max_ are all calculated parameters that correlate with each other, lurasidone plasma concentrations at each time point were chosen as independent variables to further analyze their association with somnolence using binary logistic regression.

In the first case-control study (Table 3), fed condition (OR = 3.896; p = 0.015) along with plasma concentrations at 0.25 h (OR = 2.334; p = 0.023), 0.5 h (OR = 1.234; p = 0.001), 0.75 h (OR = 1.140; p = 0.001), 1 h (OR = 1.124; p = 0.001), 1.5 h (OR = 1.088; p < 0.001), and 2 h (OR = 1.044; p = 0.003) were independently associated with an increased risk of somnolence at univariate regression. Then, all the variables showing a p ≤ 0.1 in the univariable analysis were tested by multivariable analysis. After the multivariate regression of the ‘Forward: LR’ method, only plasma concentration at 1 h (OR = 1.124; p = 0.001) was significantly associated with an increased risk of somnolence.

**Table 3:** Independent variables in the somnolence (n = 30 volunteers) and no-somnolence (n = 29 volunteers) groups and the associations between each variable and risk of somnolence in the first case-control study.

Variables	Univariate analysis		Multivariate analysis	
	OR (95%CI)	p-value	OR (95%CI)	p-value
Gender				
Male	0.857 (0.250–2.945)	0.807		
Female	1			
Age (y)	0.954 (0.900–1.012)	0.117		
Height (m)	0.302 (0.001–93.678)	0.682		
Weight (kg)	0.992 (0.932–1.055)	0.792		
Fed condition				
Fasting	1			
Fed	3.896 (1.302–11.655)	0.015*	NS	0.233
Plasma concentration at each time point				
0 h	3.737 (0.002–7389.434)	0.734		
0.25 h	2.334 (1.122–4.853)	0.023*	NS	0.245
0.5 h	1.234 (1.093–1.394)	0.001*	NS	0.077
0.75 h	1.140 (1.059–1.227)	0.001*	NS	0.105
1 h	1.124 (1.052–1.201)	0.001*	1.124 (1.052–1.201)	0.001*
1.5 h	1.088 (1.040–1.137)	<0.001*	NS	0.395
2 h	1.044 (1.014–1.075)	0.003*	NS	0.343
2.5 h	1.014 (0.995–1.033)	0.152		
3 h	1.005 (0.987–1.025)	0.574		
3.5 h	1.001 (0.979–1.023)	0.941		
4 h	0.994 (0.967–1.020)	0.632		
5 h	0.981 (0.950–1.013)	0.241		
6 h	0.969 (0.911–1.030)	0.307		
8 h	0.985 (0.815–1.189)	0.873		
12 h	1.035 (0.656–1.633)	0.881		
24 h	1.256 (0.429–3.678)	0.677		
36 h	1.908 (0.391–9.307)	0.424		
48 h	5.025 (0.735–34.353)	0.10^#^	NS	0.459

*p < 0.05; ^#^p ≤ 0.1; NS indicates no statistical significance.

In the second case-control study (Table 4), plasma concentrations at 0.25 h (OR = 1.50; p = 0.003), 0.5 h (OR = 1.072; p = 0.002), 0.75 h (OR = 1.042; p = 0.002) and 1 h (OR = 1.034; p = 0.002) were independently associated with an increased risk of somnolence at univariate regression. Variables showing a p ≤ 0.1 in the univariable analysis were then tested by multivariable analysis. After the multivariate regression using the ‘Forward: LR’ method, only plasma concentration at 0.75 h (OR = 1.024; p = 0.002) was significantly associated with an increased risk of somnolence.

**Table 4:** Independent variables in the somnolence (n = 48 times) and no-somnolence (n = 48 times) groups and the associations between each variable and risk of somnolence in the second case-control study.

Variables	Univariate analysis		Multivariate analysis	
	OR (95%CI)	p-value	OR (95%CI)	p-value
Preparation				
Reference	1			
Test	1 (0.449–2.226)	1		
Plasma concentration at each time point				
0 h	2376.793 (0.143–39601923.360)	0.117		
0.25 h	1.50 (1.146–1.962)	0.003*	NS	0.162
0.5 h	1.072 (1.026–1.121)	0.002*	NS	0.580
0.75 h	1.042 (1.016–1.070)	0.002*	1.042 (1.016–1.070)	0.002*
1 h	1.034 (1.012–1.056)	0.002*	NS	0.635
1.5 h	1.015 (0.999–1.032)	0.058^#^	NS	0.752
2 h	1.003 (0.985–1.020)	0.772		
2.5 h	0.992 (0.971–1.012)	0.430		
3 h	0.989 (0.967–1.012)	0.341		
3.5 h	0.993 (0.967–1.019)	0.581		
4 h	0.996 (0.965–1.028)	0.817		
5 h	0.980 (0.946–1.015)	0.260		
6 h	0.965 (0.905–1.029)	0.275		
8 h	0.926 (0.768–1.117)	0.421		
12 h	0.943 (0.619–1.434)	0.782		
24 h	0.902 (0.274–2.967)	0.865		
36 h	1.937 (0.422–8.891)	0.395		
48 h	2.344 (0.425–12.925)	0.328		

*p < 0.05; ^#^p ≤ 0.1; NS indicates no statistical significance.

### 3.4 Cut-off value of lurasidone concentration to predict the occurrence of somnolence

**Fig. 4:**
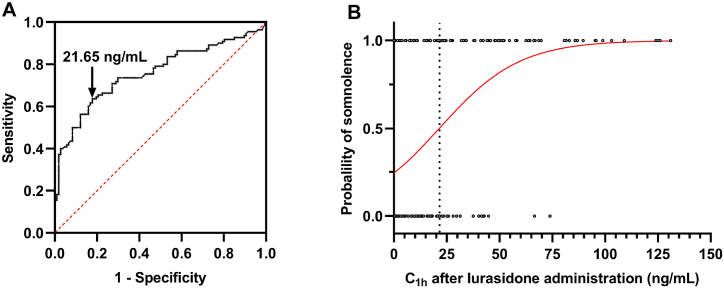
Receiver operating characteristic (ROC) curve of somnolence to determine the cut-off point for plasma lurasidone concentration 1 h after the administration of a single dose of 40 mg lurasidone hydrochloride tablet (A). Logistic regression model of lurasidone concentration 1 h after administration and probability of somnolence (B).

As described above, lurasidone plasma concentration at 1 h in the first case-control study and concentration at 0.75 h in the second case-control study were significantly associated with an increased risk of somnolence. To further explore at which time point lurasidone plasma concentration that yielded the highest combined sensitivity and specificity for predicting somnolence in all volunteers, receiver operating characteristic curves were applied. As a result, among different time points, lurasidone plasma concentration at 1 h yielded the largest AUC of 0.755 to predict somnolence in all volunteers (Fig. 4A). Moreover, 21.65 ng/mL at 1 h after administration was determined as the cut-off value that gave the maximal combined sensitivity (0.63) and specificity (0.821) for the occurrence of somnolence (Fig. 4B).

## 4 Discussion

Somnolence is a common reported side effect of antipsychotic medication [11]. In a review of antipsychotic drug-induced somnolence in several psychotic disorders, some antipsychotics were classified as high somnolence (clozapine), moderate somnolence (olanzapine, perphenazine, quetiapine, risperidone, and ziprasidone), or low somnolence (aripiprazole, asenapine, haloperidol, lurasidone, paliperidone, and cariprazine) [11]. Although positive correlations between dose and the prevalence of somnolence have been observed in some studies, such as olanzapine [27] and ziprasidone [28], when pooling all data at the same dose together, there were no consistent patterns of dose-dependent effect [11]. This speaks to different vulnerabilities in different individuals.

In this study, two independent case-control studies were performed to explore the relationship between somnolence and lurasidone exposure, along with the factors associated with an increased risk of lurasidone-induced somnolence, except for dosage. From the above results, we found that the critical risk factors associated with somnolence in two independent case-control studies were not exactly the same but very close (lurasidone plasma concentrations at 1 h and at 0.75 h), and a plasma concentration of lurasidone greater than 21.65 ng/mL at 1 h after administration was significantly predictive of somnolence in all volunteers. These findings indicate that the faster the plasma concentration of lurasidone increases by 1 h after administration in healthy volunteers, the more likely somnolence occurs. Therefore, factors leading to a rapid increase in lurasidone concentration at 1 h, such as dosage, concomitant food intake, and formulation, should be considered when prescribing the drug in clinical practice. Several studies have indicated that the dosage of lurasidone can affect the incidence of somnolence [[15], [16], [17], [18], [19]]. Fed condition was independently associated with an increased risk of somnolence in the first case-control study, which is consistent with the obvious effect of concomitant food intake on the bioavailability of lurasidone hydrochloride tablets [10]. Although lurasidone is currently available only as an immediate-release formulation, the different effects of the two quetiapine formulations (another antipsychotic) on sedation in healthy volunteers inspired us [29]. In that study, sedation was significantly greater with immediate-release quetiapine (quetiapine IR) than with extended-release quetiapine (quetiapine XR) 1 h after dosing on day 1, and quetiapine XR was associated with a lower intensity of self-reported sedation than quetiapine IR [29]. Similar differences in quetiapine XR and IR for sedation among patients with bipolar depression were also observed in another randomized, double-blind, double-dummy, parallel-group, Phase IV study [30].

The studies reported here have some limitations. First, the subjects were healthy volunteers and not patients with psychotic disorders, such as schizophrenia, who may, as a group, be of higher average weight, more likely to smoke and use substances in excess, and may follow irregular routines with respect to eating, medication intake, and sleeping. Healthy volunteers [16] as well as pediatric patients [15] subjects appear to show higher rates of lurasidone-induced somnolence when compared to the adult patient subjects in clinical studies [31]. Even information about antipsychotic drug-induced somnolence gathered from RCT studies of patients may not be generalizable to clinical practice [11]. In this study, healthy volunteers received a single dose of 40 mg lurasidone hydrochloride tablet in one period, whereas patients were required to take lurasidone daily. A second limitation is that the data were from one bioequivalence study. In one study conducted with healthy Chinese subjects, the rates of somnolence were 75% and 33.3% after a single dose of 40 mg lurasidone hydrochloride tablet and placebo, respectively [16]. Based on the result of that study [16], the minimum sample size of the test group and control group were 25 calculated using the tests for two proportions with the Power Analysis and Sample Size software (version 15, NCSS, LLC. Kaysville, Utah). Our study met these criteria, but the sample size was nevertheless, relatively small.

## 5 Conclusion

In this study, our findings indicate that the plasma concentration of lurasidone 1 h after administration effectively predicts lurasidone-induced somnolence in healthy volunteers. Despite the limitations of our findings with respect to clinical situations, this information should be helpful in designing new trials and in aiding clinicians with dosage and timing decisions.

## Author contribution statement

Hengyi Yu: Conceived and designed the study; Performed the study; Wrote the paper.

Xingxing Qi, Yinian Fang: Performed the study; Analyzed and interpreted the data.

Kaifu Wang, Donglin Zhang, Qian Chen: Analyzed and interpreted the data.

Dong Liu, Xiuhua Ren: Conceived and designed the study; Contributed analysis tools and data; Wrote the paper.

## Data availability statement

Data will be made available on request.

## Declaration of competing interest

The authors declare that they have no known competing financial interests or personal relationships that could have appeared to influence the work reported in this paper.
